# Hospital Problems in India

**Published:** 1921-01-15

**Authors:** 


### HOSPITAL PROBLEMS IN INDIA.

### Domestic Science.

The Director of Public Instruction, United Provinces, announces that one silver medal of the value of Rs. 10 will be awarded each year to the successful girl student who obtains the highest marks in domestic science in the school-leaving certificate examination; two silver medals of the value of Rs. 9 each will be awarded each year to two successful students who obtain the highest marks in domestic science in the Anglo-vernacular middle examination for girls; and that two silver medals of the value of Rs. 8 each will be awarded each year to two successful students who obtain the highest marks in domestic science in the vernacular lower middle examination for girls.

### Anthrax from Shaving-Brushes.

In connection with a death from anthrax which occurred in Coonoor, investigations conducted by the Deputy Sanitary Commissioner, Madras, showed that the deceased, who was a general merchant at Coonoor, contracted the disease and died of it by using one of a consignment of shaving-brushes supposed to be of Japanese manufacture, which he purchased from London and imported into India. A number of brushes (forty-six), forming part of this particular consignment, was traced to the Kolar Gold Fields. The brushes were taken possession of by the Health Department and a bacteriological examination of them at the Sanitary Board and the Mining Board laboratories revealed anthrax bacilli.

### Wild Animals and Snake Bites.

The annual returns of mortality from wild animals and venomous snakes in India are always interesting. In 1919 the number of persons reported to have been killed by wild animals was 2,637, or 473 more than in the previous year; while the loss of human life from snake-bite was 20,273, as compared with 22,600 in 1918. Tigers were responsible for 1,162 deaths, against 1,001; leopards claimed 469 victims; wolves, 294; bears, 118; elephants, 60; and hyænas, 33. Bihar and Orissa heads the list with the largest mortality due to the wild animal.

### General Hospital Strike.

The menial staff, consisting of ward boys and ward maids of the General Hospital at Rangoon, went on strike recently, causing considerable inconvenience and annoyance to those concerned. The strike was the result of delay in granting an increase of pay to meet the high cost of living. The strike only lasted a few days, but whilst it continued cast a heavy burden on the nursing staff.

### The Children's Hospital of Madras.

A hospital for children, known as the Lady Willingdon Hospital for Children, has recently been started in Madras, which has for its object the study and treatment of diseases peculiar to children before, during, and after birth. The institution is receiving the support of the public, the State, and the Corporation of Madras.

**The Medical Directory**, 1921. (London: T. & A. Churchill. 36s. net.)

The Medical Directory, like other books of reference, continues to grow with each issue, and users have to submit to certain eliminations in order, we presume, to present the contents of each book in as little increased a volume as possible. We regret, however, the omission from the 1921 Medical Directory (the seventy-seventh annual issue) of that useful section The Principal Laws Affecting the Profession and the Index to those Laws. Otherwise the Directory is, as usual, as complete and up to date as it is possible to make it, and as punctual in appearance as ever.

